# Modelling of the Citrus CCD4 Family Members: In Silico Analysis of Membrane Binding and Substrate Preference

**DOI:** 10.3390/ijms222413616

**Published:** 2021-12-19

**Authors:** Jorge Cantero, Fabio Polticelli, Margot Paulino

**Affiliations:** 1 Bioinformatics Area, DETEMA Department, Faculty of Chemistry, UdelaR, General Flores 2124, Montevideo 11600, Uruguay; jorgec@fq.edu.uy; 2 Research Department, Faculty of Agronomic Engineering, Universidad Nacional del Este, Ciudad del Este 7420, Paraguay; 3 Department of Sciences, Roma Tre University, 00146 Rome, Italy; 4 National Institute of Nuclear Physics, Roma Tre Section, 00146 Rome, Italy

**Keywords:** CCD4 enzymes, *Citrus clementina*, protein–membrane interactions, in silico studies, molecular dynamics simulations, carotenoids, membranes

## Abstract

Coloring is one of the most important characteristics in commercial flowers and fruits, generally due to the accumulation of carotenoid pigments. Enzymes of the CCD4 family in citrus intervene in the generation of β-citraurin, an apocarotenoid responsible for the reddish-orange color of mandarins. Citrus CCD4s enzymes could be capable of interacting with the thylakoid membrane inside chloroplasts. However, to date, this interaction has not been studied in detail. In this work, we present three new complete models of the CCD4 family members (CCD4a, CCD4b, and CCD4c), modeled with a lipid membrane. To identify the preference for substrates, typical carotenoids were inserted in the active site of the receptors and the protein–ligand interaction energy was evaluated. The results show a clear preference of CCD4s for xanthophylls over aliphatic carotenes. Our findings indicate the ability to penetrate the membrane and maintain a stable interaction through the N-terminal α-helical domain, spanning a contact surface of 2250 to 3250 Å^2^. The orientation and depth of penetration at the membrane surface suggest that CCD4s have the ability to extract carotenoids directly from the membrane through a tunnel consisting mainly of hydrophobic residues that extends up to the catalytic center of the enzyme.

## 1. Introduction

The carotenoids are isoprenoid pigments (terpenes) abundant in the membranes of all phototrophic and most heterotrophic organisms. The abundance and diversity of carotenoids in plants is due to multiple functions and properties related to their chemical structure. Carotenoids are localized in the thylakoid membranes, and their biosynthesis is carried out in chloroplasts [1].

### 1.1. Carotenoids Cleavage Dioxygenases

The Carotenoids Cleavage Dioxygenases (CCDs) constitute a big family of non-heme Fe^+2^-dependent enzymes participating in the oxidation of carotenoids and apocarotenoids, widely distributed in all living organisms [2]. These enzymes are able to catalyze an oxidative cleavage at the level of carotenoid double bonds [3]. CCDs are characterized by an N-terminal signal peptide that targets them to the chloroplast, where the peptide is cleaved off. Thus, biosynthesis and cleavage reactions occur in the chloroplast [4,5]. They are not transmembrane proteins, but they interact with membranes through a hydrophobic region that penetrates in the membrane where their substrates are located [4].

CCDs are usually very specific both in terms of the cleavage site and carotenoid substrate. All of them have a highly conserved site formed by four histidine residues that coordinate an Fe^+2^ ion.

The first CCD whose X-ray three-dimensional structure was solved is the *Synechocystis* sp. apocarotenoids cleavage oxygenase (ACO–EC 1.13.11.75) expressed in *Escherichia coli* (PDB id: 2BIW). This enzyme cuts the apocarotenoids at the position C15=C15′ [5].

A very diverse set of CCDs exists in plants [6]. The CCDs are classified by their capacity to produce ABA, a hormone implicated in the oxidative stress response and in the seeds’ dormancy. The first characterized subfamily was the one involved in the ABA biosynthesis, i.e., the 9-*cis*-epoxicarotenoids dioxygenases (NCEDs) [7,8]. Members of this family oxidize selectively at the position C11-C12 9-*cis*-violaxantine and 9-*cis*-neoxantine, giving xantoxine, an ABA precursor, as the product [9]. The functions of other subfamilies (CCD1, 2, 4, 7, and 8) are not yet fully known [6,10].

### 1.2. CCD4 Subfamily

The CCD4 subfamily is characterized by a high level of structural conservation [11]. A vast majority of CCD4s, inside the chloroplasts, might interact with the thylakoid membranes. The function of the cleavage products is not yet entirely dilucidated [6].

The CCD4 subfamily is distributed in all flowering plants [12]. The functions of the subfamily members are directly related to the colors of the fruits and flowers and their scents [13]. They are present in leaves, flowers, roots, and stems [14]. A variable number of genes coding for the CCD4 subfamily members is present in different species, with most of them having at least two CCD4 genes [6,10]. Some CCD4 genes are specifically expressed only in certain organs, and their expression can be triggered by abiotic stress [14]. Similar to other proteins of the CCD family, members of this subfamily initially possess a chloroplast targeting peptide (cpTP) in the N-terminal region [14].

They are housed inside the chloroplasts together with other enzymes of the carotenoid biosynthesis pathways, thus having easy access to their substrates [8]. They are among the most abundant proteins inside the chloroplasts, making evident their importance in the carotenoid synthesis routes in plants [15].

### 1.3. Functional Characterization of CCD4s

Most CCD4s are capable of cleaving carotenoids at the C9=C10 and C9′=C10′ positions, producing C13 apocarotenoids. However, in citrus, evidence on CCD4b indicates the ability to cleave at one of the positions C7=C8 or C7′=C8′ [14].

### 1.4. Characteristics of the CCD4s of Citrus Species

A total of eight CCD genes have been identified in citrus: two NCEDs, one CCD1, and five members of the CCD4 subfamily. The CCD4 subfamily members are named a, b1, b2, c, and d [6].

The largest number of CCD4 genes are found in citrus. The number of base pairs (bp) that make up the coding region varies from species to species and ranges from 1200 to 1800 bp [16]. The vast majority of genes do not have introns. Sequence analysis shows extensive allelic diversity, including SNPs and in-frame shift mutations [16]. The length of the predicted protein sequences ranges from 400 to 600 amino acids. The degree of amino acid sequence identity varies throughout the members of this subfamily, ranging from 30% to 98%, with typical values between 50% and 70% [6].

In oranges and mandarins, CCD4b1 was characterized as responsible for the asymmetric cleavage of β-cryptoxanthin and zeaxanthin to generate β-citraurin (C30), whose accumulation produces the intense orange-reddish color in the pigmentation of mandarin hybrids of the Fortune variety [14].

CCD4a is expressed in all tissues, but it predominates in leaves and flowers, while CCD4b1 is expressed predominantly in the peel and, to a lesser extent, in flowers [14]. CCD4c is expressed only in flowers and has been proposed as an ideal molecular marker for the study of the relationships between citrus species based on their allelic polymorphism [16]. The CCD4b2 and CCD4d are not expressed. These genes have lost important regions coding for residues conserved in the CCD family, and evidence indicates that they are pseudogenes [14].

A previous study suggested that the amino acid residues of the α-helices of citrus CCD4 have the ability to interact with membranes. This prediction was based on the amino acid sequence alone, without additional structural data being available, such as the depth of penetration or the type or orientation of the residues that would be responsible for the anchoring to the membrane [17]. On the other hand, mutational studies showed that, with the removal of the α-helical domain, the enzyme loses its ability to interact with the thylakoid membrane, leading to a loss of its enzymatic activity [7]. This evidence led us to consider the presence and location near the membrane of the α-helical domains to be essential. From this viewpoint, it must be noted that the *Zea mays* dioxygenase viviparous 14 (VP14), an enzyme active in the cleavage of 9-cis-epoxycarotenoids, is the only enzyme of plant origin with available crystallographic data that possesses the α1 and α3 domains of interaction with the membrane (PDB id: 3NPE). This is the only enzyme of plant origin with known crystallographic data that possesses the α1 and α3 domains of interaction with the membrane. VP14 is an enzyme acting on carotenoids in the 9-cis configuration and cleaving the double bond at position C11=C12 (C11′=C12′), thus participating in the first steps of the biosynthetic pathway of ABA (EC 1.13.11.51) [8].

Given the biotechnological importance of the CCD4 dioxygenases and the absence of enough data regarding their structure and mechanism of action, the present work was focused on the CCD4s from *Citrus clementina* varieties with an emphasis on those that are expressed (CCD4a, CCD4b1 (hereinafter CCD4b), and CCD4c). To this aim, in this work, a combination of different bioinformatics techniques was used to model and validate their three-dimensional structures and build the complexes with putative carotenoid ligands to elucidate substrate selectivity. Furthermore, a detailed analysis of the residues that participate in enzyme–substrate interactions, as well as in the interactions with biological membranes, was carried out using molecular dynamics simulation techniques.

## 2. Results and Discussion

### 2.1. Molecular Modeling

**Figure 1:**
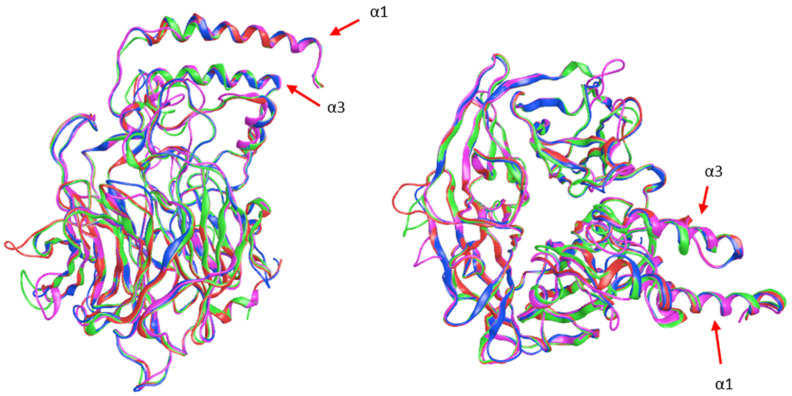
Ribbon representation of the CCD4s models: CCD4a (green), CCD4b (blue), CCD4c (pink), and VP14 (red). α-helices 1 and 3 are indicated by arrows.

The threading models of CCD4a, CCD4b, and CCD4c are shown in Figure 1, superimposed onto the three-dimensional structure of VP14, a dioxygenase from corn that possesses the α-helical domains that characterize CCDs of plant origin. These are thought to have a function in the interaction of the protein with thylakoid membranes, which is why the model that will be proposed adheres to the hypothesis that such α-helical domains interact with biological membranes in CCD4s as well.

The models obtained with PHYRE 2 display the β-propeller structure characteristic of CCDs. This structural domain is formed by seven β-sheets arranged around a central axis. At the center of this structure is located the catalytic center composed of an Fe^+2^ ion coordinated by four histidine residues, highly conserved in the members of this family.

The structural alignment between the models and the crystallographic structure of VP14 shows very small structural differences, with the root-mean-square deviation (RMSD) values ranging from 0.85 to 1.38 Å (Table 1).

The main differences in the three-dimensional structures observed in the models are found in the loops, which constitute the most mobile regions of the structures, as can be seen in Supplementary Material Figure S1.

### 2.2. Insertion of α-Helix Domains into the Membrane

**Figure 2:**
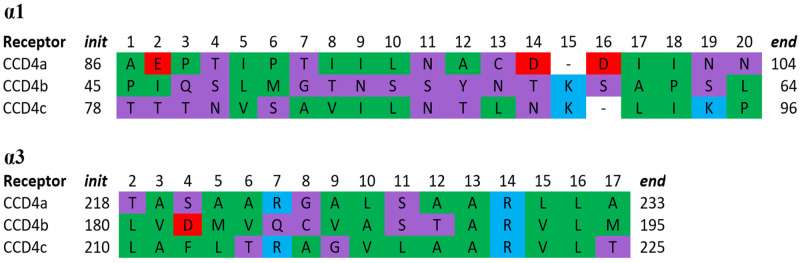
Sequence alignment of the α1 and α3 helicoidal regions of CCD4a, b, and c. Colors indicate the degree of polarity of the amino acids: red: negatively charged; blue positively charged; green: hydrophobic; violet: polar. *Init* and *end* indicate the initial and final positions of the helical regions in the sequences.

The α-helix domain of CCD4s is made up of two antiparallel α-helices, α1 and α3. In Figure 2, the alignment of the sequences of the α1 and α3 helicoidal regions for all the models is shown. It is interesting to note that, in CCD4b, the helicoidal region starts at the 45th amino acid, while, in CCD4a and CCD4c, it starts at the 86th and 78th amino acid, respectively. However, the tertiary structure of the helical domain is fairly conserved in the CCD4 subfamily members.

The α1 domain is formed by 19 to 20 amino acids, the polar ones being predominant. The α3 domain is formed by 17 mainly hydrophobic amino acids that have been predicted to interact with the hydrophobic region of the membrane. The polar and charged surfaces of the helices have been predicted to interact with the thylakoid membrane through polar interactions with the phospholipid moieties characterized by a zwitterionic structure (such as, for example, in the case of phosphatidylcholine).

It could be conjectured that the interaction of the citrus CCD4s with the thylakoid membrane through those alpha domains would allow these enzymes to directly extract the carotenoids from the membrane by orienting them to a tunnel-shaped channel, mainly formed by hydrophobic residues, that connects the membrane with the catalytic center of the enzyme.

To evaluate the way in which the amino acid residues of the α-helices of citrus CCD4s interact with the thylakoid membrane, we studied the time evolution of the depth of penetration and the contact surface extension through rigid body molecular dynamics simulations of the protein complexes with the membrane (see Section 3.4 Construction of membrane complexes for details).

The analysis was further extended through classical molecular dynamics simulations of the enzyme–substrate–membrane complexes to rationalize the experimental results and explore new hypotheses through a deeper insight of the intermolecular forces that govern protein–ligand and protein–membrane interactions and their temporal evolution. This information can help to understand the molecular bases of the CCD4–membrane interaction, the reactivity and selectivity of CCD4 enzymes with their carotenoid substrates, and the structural and dynamic behavior of these enzymes and their substrates that remained poorly explored until now.

Regarding the rigid body simulations, to refine the analysis of the interactions between the proteins and the membrane, the time evolution of hydrogen bonds through the molecular dynamics trajectory has been recorded and plotted (Supplementary Material Figure S2), and the number of hydrogen bonds was averaged. The average resulted to be nine for the CCD4a, seven for CCd4b, and ten for CCD4c. Thus, there is not a significant difference in this respect between the different proteins, indicating that the mechanism of the protein–membrane binding is similar.

**Figure 3:**
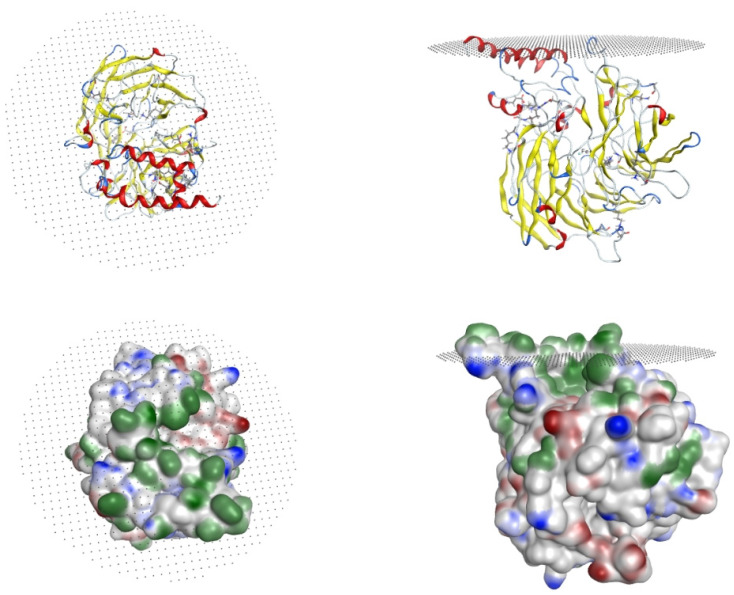
(**Top**) Ribbon representation of CCD4b model inserted in the membrane (dotted grey surface). (**Bottom**) Molecular surface representation colored by hydrophobic (green), negatively charged (red), and positively charged (blue) areas.

Figure 3 shows two representations of the CCD4b protein model inserted in the membrane following the protocol described in detail in the Methods section. A ribbon representation of the model inserted in the membrane allows to see the two α1 and α3 helices (in red) penetrate into the membrane (whose plane is represented by the grey dotted area). The protein solvent accessible surface colored by the charge distribution is also shown from two different viewpoints.

**Figure 4:**
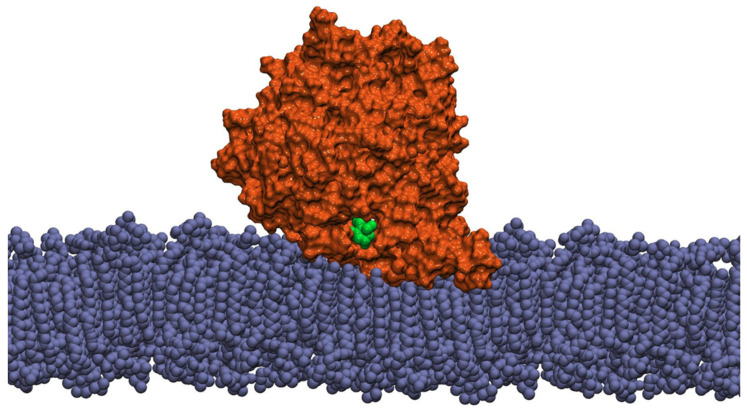
Representation of the CCD4b model (orange), including a β-carotene ligand molecule (green), interacting with a biological membrane model (blue), through its α1 and α3 domains.

The penetration of the protein into the thylakoid membrane is such that the hydrophobic groups of the α-helices interact with the fatty acids inside the membrane (Figure 4). Depending on the protein subtype, the initial phospholipid membrane penetration was from 5.2 to 7.0 Å, with a predicted average ΔG_transfer_ value of −58.1 kJ/mol (Table 2). These values are consistent with those observed for VP14, with a reported penetration depth of 7 Å [8]. Thus, the proposed model suggests that the CCD4s are putative peripheral membrane proteins.

A comparison among all the CCD4–membrane models gives evidence of a similar placement inside the membrane. In Table 2, values of the angle, depth, and energy of transfer from the solvent to the membrane are reported.

It is evident from the values reported in Table 2 that all the CCDs are predicted to interact with the membrane in a similar manner.

The penetration of the hydrophobic region formed by the α-helices of the CCD4s places the substrate channel in proximity of the thylakoid membrane, allowing to hypothesize that the enzyme can have direct access to the membrane interior. In this regard, it is important to note that carotenoids are among the most abundant constituents of the thylakoid membrane.

### 2.3. Analysis of the Energetic Components and Thermodynamic Equilibrium

In Table 3 are reported the averaged values and the corresponding standard deviations of the van der Waals and electrostatic components of the interaction energy, as well as the value of the total interaction energy (Uab) obtained from the analysis of the classical molecular dynamics trajectories of the complexes formed by CCD4a, b, and c with selected carotenoids.

Carotenoids are highly hydrophobic molecules. The enzymes’ binding site is a highly hydrophobic linear channel as well, favoring interactions with the ligand that are governed by short-range VDW-type interactions. Polar interactions are also present, and the contribution to the interaction energy is greater in xanthophylls as compared to aliphatic carotenes. The xanthophylls in their polar regions (sites characterized by the presence of hydroxyl groups) tend to form hydrogen bonds with water molecules or interact with other polar molecules of the system.

The evolution of the interaction energy of CCDb in the membrane with a carotenoid substrate has been represented as a function of the trajectory time, indicating an equilibration regime (Figure S3).

### 2.4. Structural Analysis: RMSD and the Root Mean Square Fluctuation (RMSF).

The RMSD values were plotted as a function of the simulation time for complexes formed by CCD4a, b, and c models and the seven carotenoids under study (see Figure S4 in Supplementary Material). This plot demonstrated that the structural stability was reached by 5 ns of simulation for all three proteins in all the carotenoid complexes.

**Figure 5:**
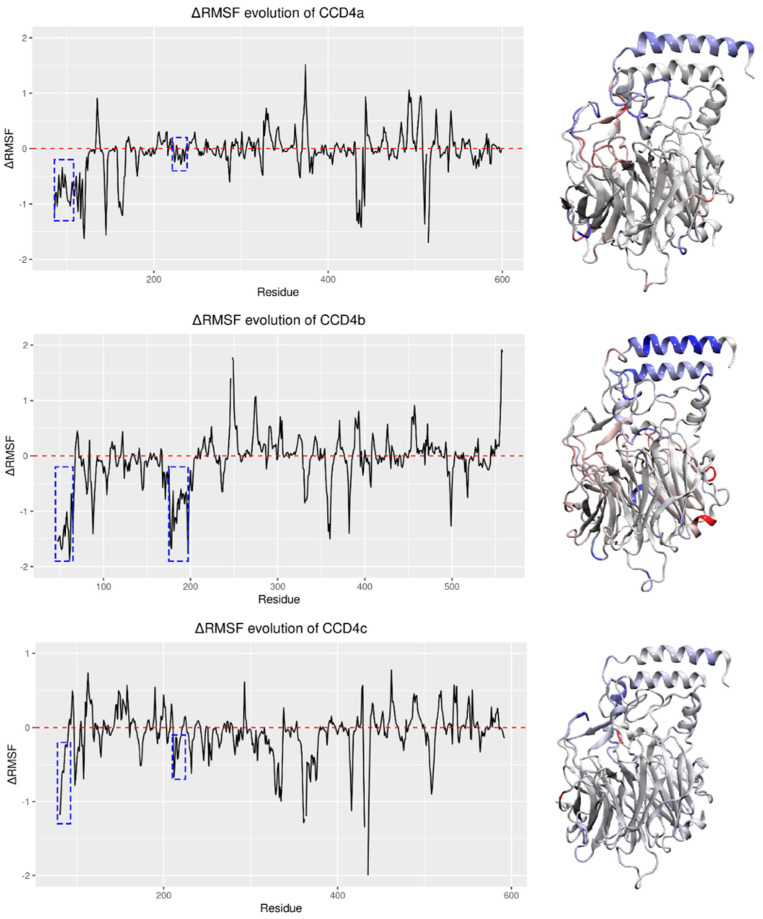
Left panels: ΔRMSF values for CCD4a, CCD4b, and CCD4c (for details, see text). The first blue dotted squares from the left indicate the sequence region corresponding to the α1 helices. The second blue dotted squares indicate that corresponding to the α3 helices. Right panels: three-dimensional structures of the models. More mobile regions, corresponding to ΔRMSF > 0, are colored in red. Less mobile regions (ΔRMSF < 0) are colored in blue.

The α1 and α3 helical domains, having a direct interaction with the membrane, are the regions most affected by the presence of the membrane, evidenced by a decrease in the average fluctuations (negative ΔRMSF values) (Figure 5). It is worthwhile to note that the α1 helix is characterized by a lower mobility (more intense blue color) with respect to the α3 helix. Regarding the regions whose mobility is increased (higher ΔRMSF values and red colored in Figure 5, right panels), these are loops exposed to the stroma liquid matrix of the chloroplast.

### 2.5. Carotenoids’ Distances from the Fe^+2^ Atom and Binding Energy Values

Binding energy values between CCD4a, b, and c and all the carotenoids under study were evaluated from the molecular dynamics simulations as a time average. The averaged values of the MM/PBSA binding free energy ΔG_binding_ and the corresponding standard deviation values estimated for the structures extracted from the equilibrated molecular dynamics trajectories are reported in Table 4.

As predicted, in line with experimental evidence, the best CCD4b substrate β-cryptoxanthin (RRX) displays the lowest binding free energy, confirming the reliability of the structural model and of the simulation approach.

Another observation that emerges from the analysis of Table 4 (distances highlighted in grey) is that the MD simulations indicate that the anchorage and the catalytic event is mainly centered around a region near the carotenoid C7=C8 and C9=C10 double bonds, suggesting that the catalysis occurs in an asymmetric fashion.

The different degrees of polarity of the different carotenoids are probably at the root of their different positioning near the catalytic ion. Apolar carotenoids likely establish different interactions with the enzymes’ active site with respect to polar ones that display hydroxylated b-rings. This property has an impact on the positioning of the substrate in the active site and, as a consequence, results in the positioning of a different double bond near the catalytic site.

When the MM/PBSA binding energy values are considered together with the distance values observed, it can be concluded that the tendency of the catalytic site of oxidases is to position the carotenoids C9=C10 double bond near the Fe^+2^ ion, promoting in this way an asymmetric cleavage at this site.

With respect to substrate specificity, the data indicate that, in the case of CCD4a, an asymmetric oxidation in C9=C10 of the β-carotene as the best putative substrate would be favored. In fact, this compound displays the best binding energy of −162.54 kJ/mol, highlighting a certain degree of carotenoid specificity for CCD4a.

In the case of CCD4c, if we take into account the distances between the carbon atoms of the putative substrates and the Fe^+2^ ion in the catalytic site, an asymmetric (C9=C10) as well as a symmetric (C15=C15′) cleavage should be favored. In this case, β-cryptoxanthin is predicted to be the best substrate, with a binding energy of −128.21 kJ/mol.

Special attention was devoted to the CCD4b enzyme in which the binding energy values point to β-cryptoxanthin as the best ligand. As a clear result was not obtained from the 20 ns MD trajectory with respect to the cleavage site, a 1 μs trajectory was carried out, whose results are presented in Table 4 and indicated as RRX*. These results confirmed β-cryptoxanthin to be the best ligand and indicated that CCD4b catalyzes an asymmetric cleavage of β-cryptoxanthin.

**Figure 6:**
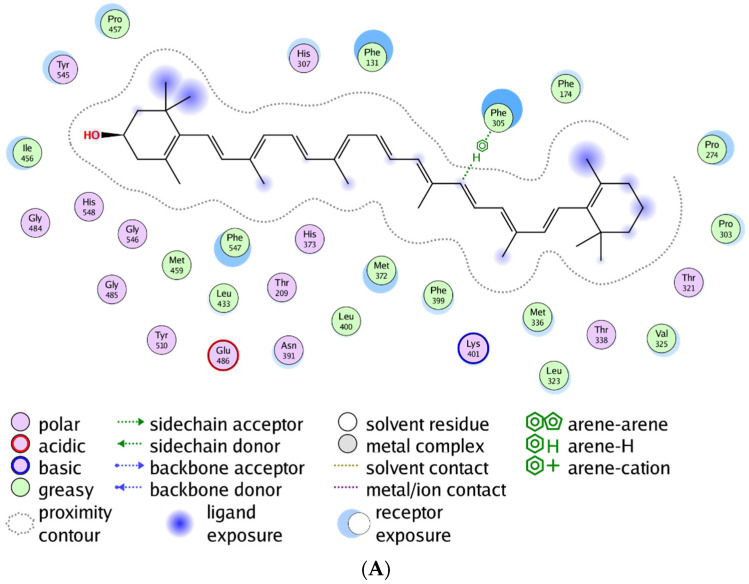

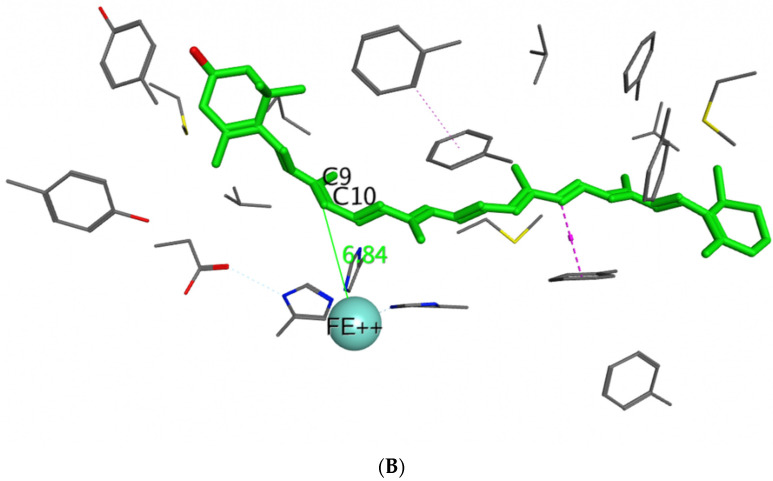
(**A**) 2D representation of the interactions established by β-cryptoxanthin within the CCD4b catalytic site. Only the residues contacting the carotenoid at less than 4.5 Å distance are shown. (**B**) 3D representation of the interactions in which the catalytic Fe^+2^ ion is represented by a cyan sphere.

To analyze in deeper detail how RRX is positioned in the catalytic site of CCD4b and the interactions taking place, the ligand interaction tool of MOE was used. The results of this analysis, shown in Figure 6, indicate that the main interactions are established with the C9=C10 double bond.

### 2.6. Analysis of Membrane–Protein Interaction

**Figure 7:**
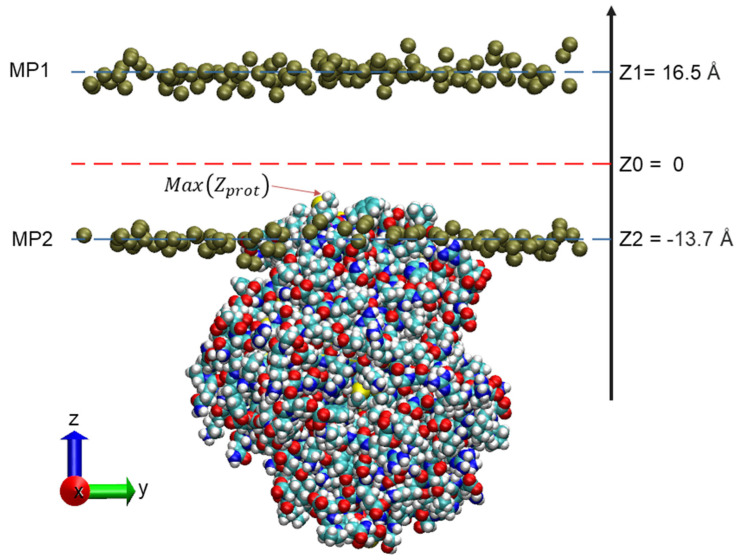
Three-dimensional view of the CCD4b inserted into the membrane. The phosphate groups (dark yellow balls) were aligned in the XY plane and projected on the Z axis. The first set MP2 includes all phosphate groups on the membrane side in which the protein was inserted. The second set MP1 includes the phosphate group belonging to the other layer of the membrane. Max(Z_prot_) is the maximum atomic position observed for the protein along the Z axis for the 20 ns trajectory (not considering hydrogen atoms). Z1 and Z2 are the average values of the phosphate groups’ Z coordinates belonging to each layer.

The model of CCD4b inserted in the membrane, at the end of the 20 ns molecular dynamics trajectory, is displayed in Figure 7.

**Figure 8:**
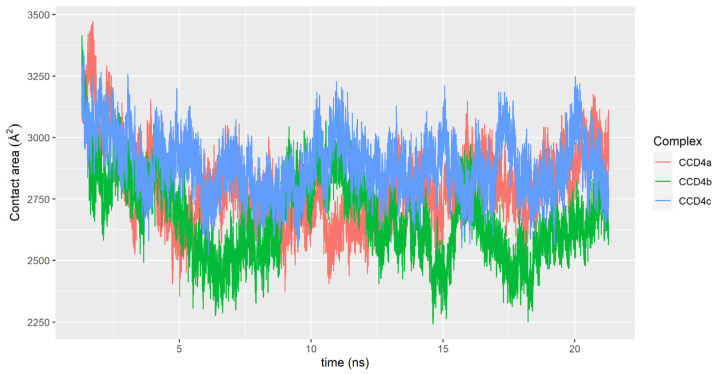
Evolution of the contact surface area (Å^2^) in the complexes formed by CCD4a (red), CCD4b (green), and CCD4c (blue) and the membrane. CCD4a displays a mean value of 2797.15 (±153.89) Å^2^, CCD4b of 2674.08 (±163.75) Å^2^, and CCD4c of 2885.87 (±118.13) Å^2^.

All the area involved in the contact as well as the membrane thickness, protein depth, and relative protein depth in the membrane were analyzed. The area involved in the contact observed throughout the molecular dynamics trajectory between the different proteins and the membrane indicates that the interaction is significant and covers a non-negligible surface that typically ranges from 2250 to 3250 Å^2^ (Figure 8).

This contact surface is mainly dominated by residues of the α-helical domain, as was observed in the structure of VP14 by Messing et al. [8]. This allows us to suggest the same protein–membrane interaction mechanism for citrus CCD4s, previously pointed out in the studies by Ma et al. [17]. These results indicate that a stable interaction, due to the orientation and penetration on the surface of the thylakoid membrane, would enable CCD4s to extract the membrane-soluble carotenoids through a tunnel formed mainly by hydrophobic residues that extends up to the catalytic center of the enzymes, as also was hypothesized before by our research group [19]. The α1 and α3 helical domains included in these models have been found to be essential for the interaction with thylakoid membranes, which promotes a decrease in their movements. These domains are characterized by an abundance of nonpolar amino acids that favor the interaction with the membrane, allowing a penetration of approximately 10 Å, which constitutes a relative penetration of approximately 30 to 35% of the membrane width (Supplementary Materials, Figures S5–S7).

## 3. Materials and Methods

### 3.1. Proteins’ Structural Model Building

#### 3.1.1. Modelling by Threading

For the construction of structural models of the CCD4a, CCD4b, and CCD4c proteins, including the α1 and α3 domains that interact with the thylakoid membranes of the chloroplast and are present only in proteins of plant origin, the software PHYRE2 (Protein Homology/analogY Recognition Engine) was used [20]. PHYRE2 combines the threading/ab initio modeling method with the modeling of parts of the sequence by homology with multiple templates.

Some structural elements of a model previously published by our group were used [19] given their established validity. In this sense, after an inspection of the Fe^+2^ coordination sphere, the position of Fe^+2^ was defined by superimposing both models, verifying the Fe^+2^ coordination sphere with the histidine residues (His257, His307, His373, and His548 in the CCD4b1).

The models obtained for CCD4a, CCD4b, and CCD4c were compared with the structure of the *Zea mays* dioxygenase viviparous 14 (VP14), an enzyme active in the cleavage of 9-cis-epoxycarotenoids (PDB ID: 3NPE). The resolution of the crystal structure is 3.2 Å, with an R-factor = 0.242 and an R-free = 0.272.

#### 3.1.2. Refinement and Molecular Dynamics

All calculations were carried out using NAMD2 version 2.12 software optimized to work on CUDA cores [21].

The force field selected for the simulation was the Charmm36 natively compatible with the NAMD2 program [22]. The parameterization of the ligands was carried out through the CGenFF (CHARMM General Force Field) program, compatible with the Charmm36 force field [23]. In this program, the assignment of parameters and charges is done by analogy, studying the connectivity patterns and the chemical environment of each atom. For the Fe^+2^ atom in coordination, the unbound scheme was used. The Fe^+2^–protein interaction was modeled using electrostatic and VDW terms, thus allowing the study of possible changes in the coordination center. The VDW interactions were modeled by a 12-6 Lennard-Jones (LJ) potential following the Charmm36 force field model. The parameters R_min_ and ε were obtained from Li et al. [24] and were optimized for this system.

The validation of the models was carried out by means of molecular dynamics simulations, for which a water box with explicit solvent was built defining 15 Å of margin between the outermost atom and the edge of the box in each direction, with the dimensions of the periodic cells of 88 × 100 × 86 Å. NaCl counter ions were used to ensure the overall charge neutrality of the system. The water boxes were built using the TIP3P water models, and the water molecules were considered rigid bodies, allowing their rotation and translation along the box.

For all simulations, systems under periodic boundary conditions were considered, with a time step of 2 fs. The sampling stages were carried out every 2 ps. The electrostatics of the system was treated using the Particle Mesh Ewald (PME) method with a grid size of 1 Å and a cutoff distance for long-range interactions of 12 Å.

Each simulation consisted of four stages:

- Refinement through energy minimization with 2000 steps using conjugated gradients and applying harmonic restraints on the main chain atoms of the protein and on the Fe^+2^ coordination sphere to avoid possible deformations in the initial stages.
- Heating stage from 0 to 300 K in 100 ps simulation time, keeping the pressure constant at 1 atm and the harmonic restraints on the main chain atoms and the coordination sphere of the Fe^+2^ ion.
- Molecular dynamics simulation stage at a constant temperature of 300 K for 1 ns, maintaining the harmonic restraints of the previous stages.
- Molecular dynamics simulation stage at constant temperature (300 K) for 20 ns, without harmonic restraints.

### 3.2. Construction of the CCD4x–Ligand Complexes

Given the structural similarity between the CCD4a, CCD4b, and CCD4c structural models obtained by threading with those obtained by homology, the initial position of the ligands in the active site was established by structurally aligning both models and taking the position of the carotenoid modelled in previous works of our research group [19]. All complexes were validated by molecular dynamics simulations. Figure S8 shows structural formulas and numbering of the ligands here studied (apocarotenoid 3-hydroxy-8′-apocarptenol (3ON), α-carotene, β-carotene, lutein, lycopene, β-cryptoxanthin, and zeaxanthin).

### 3.3. Molecular Dynamics Simulation of the Complexes

The molecular dynamics simulation of the complexes was performed following the protocol described above in the Refinement and Molecular Dynamics section. The simulation was divided into four stages: energy minimization, heating from 0 to 300 K in 100 ps, equilibrium dynamics at 300 K for 1 ns, restricting the position of the main chain atoms, and production. Then, triplicated sampling trajectories (without any type of restriction) of 20 ns were obtained, and statistical sampling of the physicochemical parameters was evaluated.

### 3.4. Construction of Membrane Complexes

#### α-. Helical Domains and Their Interaction with Membranes

To propose the spatial arrangement of protein structures in lipid bilayers, we used the OPM (Orientations of Proteins in Membranes) program [25], in which each protein is considered as a rigid body that can freely interact with a hydrophobic layer of adjustable width. The orientation of the protein is optimized by a rigid body molecular dynamics simulation, always considering the protein as the solute whose relative position with respect to the membrane must be optimized by means of an implicit (non-structural) representation. As a first step, the free energy of protein transfer to the membrane from an aqueous solution medium (ΔG_transfer_) is minimized by a calculation at different penetration levels, moving the protein at a speed of 0.2 Å/step. Finally, the optimal penetration depth, the angle of rotation of the center of mass with respect to the plane formed by the hydrophobic layer, the residues in contact with the membrane and the residues outside it, and the optimal value of the ΔG_transfer_ are reported.

For the construction of the membrane, we used a pre-equilibrated membrane model of phosphatidylcholine (POPC) that initially covered a surface area of 102 × 140 Å. The initial thickness, measured as the distance between the bilayer phosphates, was 39 Å for the three CCD4a, b, and c models. The protein structures were coupled to the membrane layers, respecting the depth and orientation obtained in the previous step and removing the POPC units that overlapped with the protein.

The model of the protein–membrane complexes was subjected to molecular dynamics simulations following the protocol described above in the Refinement and Molecular Dynamics section, keeping the surface of the membrane constant (formed by the XY plane). The simulation was divided into four stages: energy minimization, heating from 0 to 300 K in 100 ps, equilibrium dynamics at 300 K for 1 ns restricting the position of the main chain (main chain) atoms, and a production of all the complexes models was obtained with triplicated trajectories of 20 ns.

Finally, a one microsecond (1 µs) trajectory was obtained for the CCD4b-β-cryptoxanthin complex to model a more biologically realistic event considering that this is one of the substrates reported in literature [14].

### 3.5. Analysis of Molecular Dynamics Simulations

#### 3.5.1. Ligands Interaction Energy (Uab)

The ligand interaction with the modelled macromolecular system (protein, solvent, and ions) has been evaluated through molecular dynamics simulation according to the Equation (1):

(1) ⟨Uab⟩=⟨U−Ua−Ub⟩

where

U is the total potential energy of the system,
Ua is the potential energy of receptor, ions, and solvent (ligand was excluded),
Ub is the potential energy of the ligand,
Uab is composed by non-covalent energy partition and is split in the van der Waals (VDW) and the electrostatic (ELECT) components.

#### 3.5.2. Protein–Membrane Interactions

For the study of the influence of the membrane on the CCD4x domains throughout the molecular dynamics simulations, the residues that participate directly in the interaction, the temporal evolution of the contact surface area between the protein and the membrane, and the characterization of the non-bonded interactions, such as the pattern of hydrogen bonds, the electrostatic, and VDW components, were analyzed.

In addition, changes in the membrane structure related to the insertion of these proteins have been studied.

The calculation of the mobility of the residues that make up a protein is a very important measure of its stability; one of the ways to represent it is the mean square fluctuation or RMSF (root-mean-square fluctuation).

The differences between the root-mean-square fluctuations (ΔRMSF) of the CCD4x models solvated or inserted in the membrane were calculated following Equation (2):

(2) $$\Delta {\mathrm{RMSF}}_{i}=\langle {\mathrm{RMSF}}_{i}\rangle {}_{\mathrm{memb}}-\langle {\mathrm{RMSF}}_{i}\rangle {}_{\mathrm{sol}}$$

where

$\langle {\mathrm{RMSF}}_{i}\rangle {}_{\mathrm{memb}}$ is the value of the root-mean-square fluctuation of residue i in the membrane interaction model,
$\langle {\mathrm{RMSF}}_{i}\rangle {}_{\mathrm{sol}}$ is the value of the root-mean-square fluctuation of residue i in the solvent model.

A set of in-house-developed algorithms was used to estimate three parameters in relation to the penetration of the protein inside the membrane throughout the simulation, i.e., the time evolution of membrane thickness, the depth of penetration of the protein inside the membrane, and the relative membrane penetration of the protein.

To measure the penetration depth of the protein into the membrane, the phosphate groups aligned in the XY plane and projected on the Z axis were grouped in two sets: a first set, MP2, is defined by all phosphate groups on the membrane side in which the protein was inserted. The second set, MP1, is defined by the phosphate groups belonging to the other layer of the membrane.

The average thickness of the membrane was calculated according to Equation (3):

(3) $${\mathrm{Thickness}}_{t}=\frac{\sum_{i}{\left(Z_{\mathrm{MP}1\left(i\right)}\right)}_{t}}{n_{\left(i\right)}}-\frac{\sum_{j}{\left(Z_{\mathrm{MP}2\left(j\right)}\right)}_{t}}{n_{\left(j\right)}}\;$$

where

$Z_{\mathrm{MP}1\left(i\right)}$ is the set of values on the Z axis of the cartesian coordinates of the i phosphates that belong to the set MP1 at time t,
$Z_{\mathrm{MP}2\left(j\right)}$ is the set of values on the Z axis of the cartesian coordinates of the j phosphates that belong to the set MP2 at time t,
$n_{\left(i\right)}$ is the number of phosphates belonging to the MP1 set,
$n_{\left(j\right)}$ is the number of phosphates belonging to the MP2 set.

The depth of penetration of the protein inside the membrane has been calculated using Equation (4):

(4) $${\mathrm{Depth}}_{t}=\mathrm{Max}{\left(Z_{\mathrm{prot}}\right)}_{t}-\frac{\sum_{j}{\left(Z_{\mathrm{MP}2\left(j\right)}\right)}_{t}}{n_{\left(j\right)}}\;$$

where

$\mathrm{Max}{\left(Z_{\mathrm{prot}}\right)}_{t}$ is the observed maximum value of the atomic cartesian Z coordinate of the protein (not considering hydrogen atoms).

Given that the thickness of the membrane is variable through the simulation, the relative membrane penetration of the protein (RPD) through all the simulations was evaluated using Equation (5):

(5) $${\mathrm{RPD}}_{t}=\frac{{\mathrm{Depth}}_{t}}{{\mathrm{Thickness}}_{t}}*100$$

#### 3.5.3. Calculation of Binding Free Energy Using the MM/PBSA Method

The conformational sampling for MM/PBSA binding free energy calculation was carried out on the trajectories generated during the equilibrium molecular dynamics. Three energy contributions were calculated following the MM/PBSA method. First, in the gas phase, the energy difference between the complex and the sum of the energies of the receptor and the ligand separately was calculated using NAMD and the Charmm36 force field (MM). Then, the polar contribution to the free energy of solvation was calculated numerically using the Poisson–Boltzmann (PB) equation implemented in the APBS (Adaptive Poisson–Boltzmann Solver) program [26]. Subsequently, the changes (differences) of the solvent accessible surface area (SASA) were measured, and, from them, the nonpolar contribution to the free energy of solvation was estimated through the linear relationship with SASA. Finally, the binding free energy ΔG_binding_ was estimated by the following Equation (6):

(6) $$\Delta G_{\mathrm{binding}}=\Delta H-T\Delta S=\langle \Delta {\mathrm{MM}}_{\mathrm{gas}}+\Delta G_{\mathrm{polar}}+\Delta G_{\mathrm{nonpolar}}-T\Delta S\rangle$$

where

H is the enthalpy,
T is the absolute temperature,
S is the entropy of the system,
$\Delta {\mathrm{MM}}_{\mathrm{gas}}$ is the energy difference, calculated by molecular mechanics, between the complex and the sum of the energies of the receptor and the ligand separately, in the gas phase,
$\Delta G_{\mathrm{polar}}$ is the polar contribution to the free energy of solvation,
$\Delta G_{\mathrm{nonpolar}}$ is the non-polar contribution to the free energy of solvation,
TΔS is the entropic contribution to the free energy.

The group of compounds studied is structurally similar as they all belong to the structural class “carotenoids”. It can be assumed that their contribution to the differences in entropy is similar and that, therefore, the changes in free energy due to the entropic factors cancel each other out when the differences between the free energies are calculated (expressed as ΔΔG). As a consequence, the TΔS term can be ignored as usual in the MMBPSA methods.

## 4. Conclusions

Novel three-dimensional models of the members of the CCD4 family (a, b, and c) from *Citrus clementina*, including α-helix domains that interact with the membrane, have been constructed in order to study their interaction with thylakoid membranes. The models have been validated through molecular dynamics simulations in water, and all of them have displayed structural stability throughout the simulation trajectories.

Evidence is provided that the α1 and α3 helices’ domains included in these models are essential for the interaction with thylakoid membranes and that, in their presence, the average fluctuation in these domains decreases, stabilizing the structure. A protein penetration of 10 Å inside the membrane constitutes a relative penetration of approximately 30–35% to the membrane.

The region that involves α1 is characterized by the domain of polar amino acids that interact with the polar groups of the membrane. Moreover, α3 is characterized by the abundance of apolar amino acids that favor interaction with the hydrophobic core of the membrane. All in all, it can be hypothesized that the behavior of the three proteins in their interaction with the membrane is similar, even considering that there are differences in the total number of polar and charged amino acids in the α1 domain. This hypothesis is reinforced by the similar behavior observed for the three proteins in the analysis of the proteins’ membrane penetration depth and of the mobility of the helical domain in the interaction with the membrane region. A further confirmation comes also from the observation of a similar hydrogen pattern formed by the three proteins with the membrane.

According to the interaction energy Uab values, a preference of the enzymes is observed for the binding of xanthophylls (ZEX, LUT, and RRX). When the binding energy is calculated using the more sophisticated MM/PBSA method, the averaged values of binding free energy ΔG_binding_ indicate a similar tendency; as well, carotenes, such as BCR, are proposed as putative substrates. These results confirm what is known from the experimental data, i.e., that both xanthophylls and carotenes bind to CCD4 [6].

Finally, the putative site of oxidative cleavage shows a clearly asymmetric tendency at positions C7=C8 (C7′=C8′) and C9=C10 (C9′=C10′), a result consistent with other already known members of the CCD4 type [6,14].

## Figures and Tables

**Table 1:** RMSD values (lower diagonal) and percentage of sequence similarity (upper diagonal, colored in violet) calculated for the CCD4a, CCD4b, and CCD4c models as compared to the crystal structure of VP14. The sequence alignment was obtained with the BLOSUM62 substitution matrix (gap open penalty = 7, gap extension penalty = 1) using the MOE-Align program (Molecular Operating Environment [18]).

RMSD/Similarity	CCD4a	CCD4b	CCD4c	VP14
CCD4a	--	62.70	72.00	51.90
CCD4b	1.14	--	64.30	52.60
CCD4c	1.38	0.85	--	49.60
VP14 (X-Ray)	0.95	0.69	0.85	--

**Table 2:** Values of the angle of inclination of the proteins’ first principal axis of inertia with respect to the membrane plane, of the depth of penetration in the membrane, and of the free energy of transfer from the solvent to the membrane (ΔG_transfer_).

Protein	Angle	Depth (Å)	ΔG_transfer_ (kJ/mol)
CCD4a	32° (±4°)	7.0 (±0.6)	−64.4
CCD4b	39° (±4°)	5.8 (±1.3)	−61.5
CCD4c	28° (±4°)	5.2 (±0.5)	−48.5

**Table 3:** Values of the total interaction energy (Uab) and van der Waals and electrostatic components averaged for all complexes. Average values are evaluated as a function of time for all trajectories. Best interaction energy values are highlighted in grey. All values are in kJ/mol. Abbreviations 3ON: 3-hydroxy-8′-apocarptenol; ACR: α-carotene; BCR: β-carotene; LYC: lycopene; LUT: lutein; RRX: β-cryptoxanthin; ZEX: zeaxanthin.

Receptor	Ligand	Uab (ELECT) Mean (±SD)	Uab (VDW) Mean (±SD)	Uab (Total) Mean (±SD)
CCD4a	3ON	−116.50 (±23.93)	−274.37 (±16.58)	−390.87 (±23.61)
	ACR	−33.11 (±10.16)	−344.21 (±18.75)	−377.32 (±22.07)
	BCR	−21.03 (±8.75)	−344.23 (±17.66)	−365.26 (±19.51)
	LYC	−32.66 (±10.96)	−351.83 (±15.51)	−384.49 (±18.94)
	LUT	−110.95 (±21.48)	−342.63 (±17.54)	−453.58 (±23.83)
	RRX	−77.24 (±16.85)	−333.07 (±17.81)	−410.30 (±21.77)
	ZEX	−135.94 (±25.19)	−316.85 (±18.62)	−452.79 (±26.42)
CCD4b	3ON	−124.99 (±27.10)	−271.27 (±18.53)	−396.27 (±24.13)
	ACR	−33.41 (±10.80)	−351.57 (±16.37)	−384.97 (±21.24)
	BCR	−31.76 (±10.65)	−342.07 (±14.83)	−373.83 (±18.16)
	LYC	−32.05 (±10.86)	−367.96 (±15.46)	−400.02 (±19.02)
	LUT	−119.87 (±23.65)	−331.35 (±17.78)	−451.22 (±24.61)
	RRX	−76.22 (±12.89)	−350.70 (±15.71)	−426.93 (±20.45)
	ZEX	−121.58 (±25.71)	−348.27 (±18.43)	−469.85 (±26.72)
CCD4c	3ON	−137.52 (±25.04)	−257.66 (±16.61)	−395.18 (±24.23)
	ACR	−29.88 (±10.07)	−355.33 (±14.78)	−385.21 (±17.95)
	BCR	−32.91 (±11.40)	−346.46 (±16.16)	−379.36 (±21.21)
	LYC	−36.42 (±11.65)	−363.98 (±17.23)	−400.40 (±19.95)
	LUT	−137.28 (±28.76)	−318.32 (±18.68)	−455.60 (±26.78)
	RRX	−83.85 (±18.59)	−329.94 (±18.81)	−413.79 (±23.63)
	ZEX	−132.90 (±23.44)	−360.72 (±18.69)	−493.62 (±24.86)

**Table 4:** Values of the distances between the C7, C8, C9, C10, C15, and C15′ carbon atoms of each carotenoid and the Fe^+2^ ion. Lower distance values are highlighted in grey. In the last column, binding free energy values, evaluated by the MM/PBSA method, are reported in kJ/mol units. Best binding energy values are highlighted in green. RRX* indicates the results obtained by extending the MD trajectory of the CCD4b–RRX complex to 1 μs.

Receptor	Ligand	C7	C8	C9	C10	C15	C15′	MM/PBSA (±SD)
CCD4a	ACR	9.3	8.7	8.4	8.2	10.6	11.9	−148.50 (±14.88)
	BCR	8.2	7.1	6.3	6.0	8.0	9.2	−162.54 (±17.60)
	LUT	9.5	8.5	8.6	8.3	11.2	11.9	−136.63 (±17.78)
	RRX	7.4	7.3	7.3	8.4	10.9	11.2	−133.16 (±18.15)
	ZEX	7.8	7.04	6.5	7.4	10.2	11.1	−124.20 (±17.85)
CCD4b	ACR	11.8	12.3	12.2	11.4	11.7	11.7	−123.18 (±17.91)
	BCR	11.1	11.2	10.7	9.5	9.4	10	−142.60 (±19.08)
	LUT	7.5	7.1	6.5	7.3	9.5	10.6	−119.18 (±29.12)
	RRX	10.1	9.9	8.9	7.8	5.8	6.8	−150.19 (±16.83)
	RRX*	8.9	8.0	6.8	6.9	7.9	8.6	−164.57 (±15.74)
	ZEX	7.0	6.7	6.2	7.1	10	11	−88.43 (±22.31)
CCD4c	ACR	12.3	12	11.3	10	7.9	7.3	−106.32 (±19.08)
	BCR	10.9	9.9	8.5	8.2	5.8	6.3	−92.58 (±27.12)
	LUT	11.4	12.2	11.9	11.6	11.6	11.3	−105.77 (±28.35)
	RRX	10.8	11.2	10.5	9.8	8.7	9.12	−128.21 (±20.68)
	ZEX	12.2	11.14	9.93	8.8	6.4	6.8	−99.74 (±19.03)

## Data Availability

All data are available by request.

## Supplementary Materials

The following are available online at https://www.mdpi.com/article/10.3390/ijms222413616/s1.
