# Supplementary material for: Modelling of the Citrus CCD4 Family Members: In Silico Analysis of Membrane Binding and Substrate Preference
Source: Int J Mol Sci. 2021 Dec 19;22(24):13616. doi: 10.3390/ijms222413616 (PMC8708828; doi:10.3390/ijms222413616)
Supplement: Supplementary file 1 [file ijms-22-13616-s001.zip › ijms-1493082-supplementary.pdf]

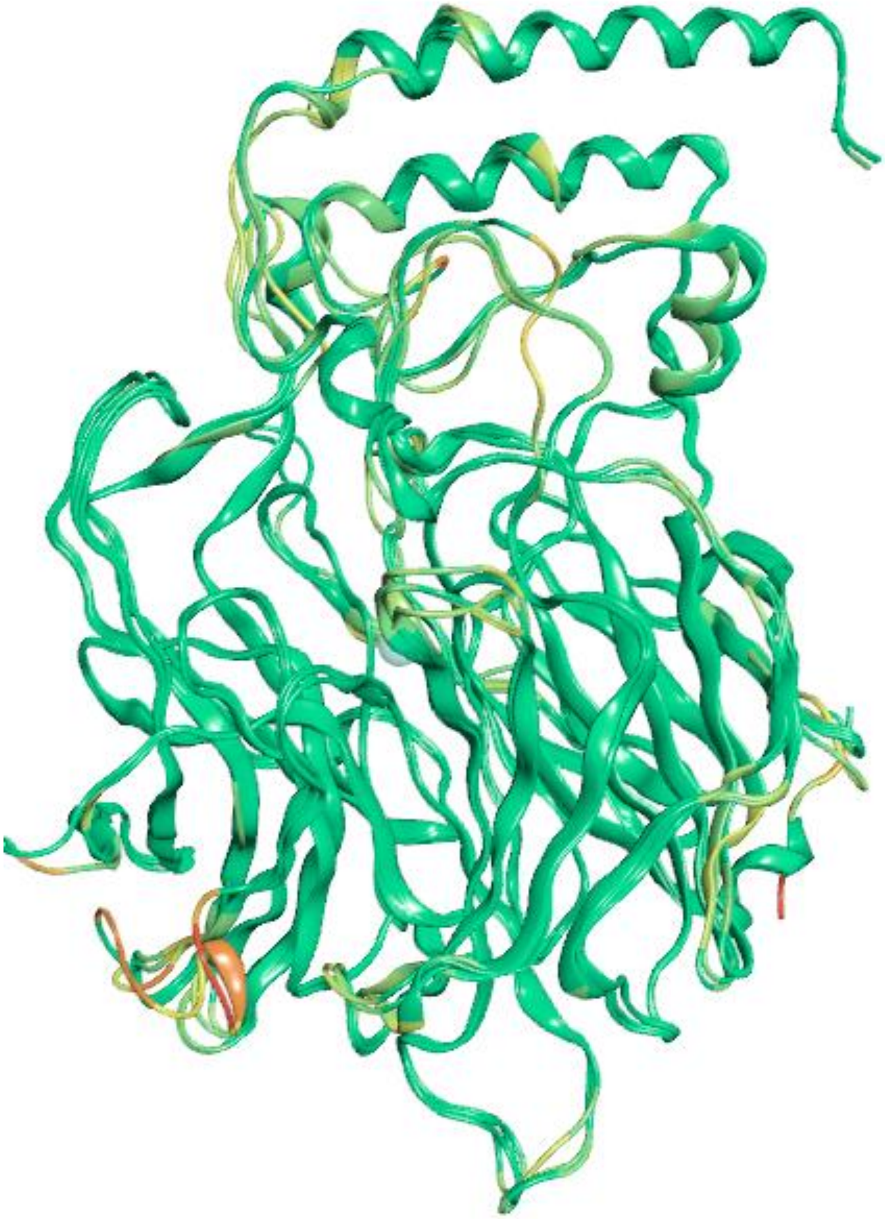

Figure S1. Ribbon representation of the models obtained for CCD4a, CCD4b, CCD4c and of the three-dimensional structure of VP14 colored according to RMSD values in the range 0.0-1.5 Å. Low values (green), medium values (yellow) and high values (red).

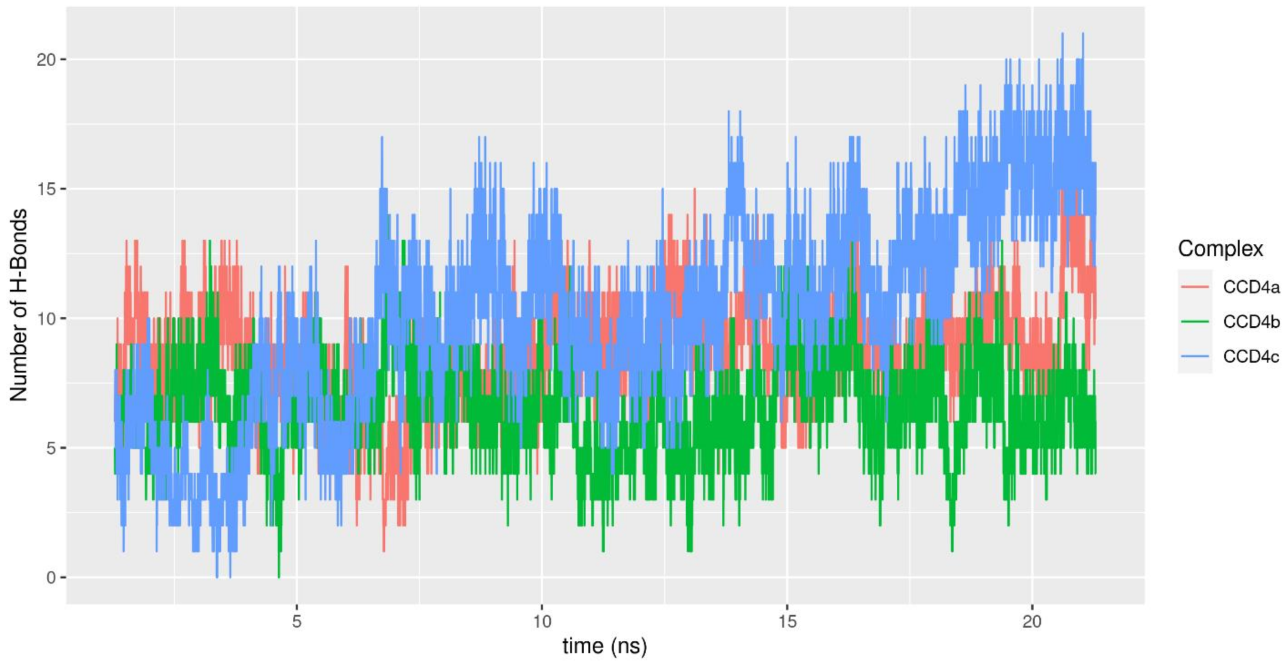

Figura S2. Temporal evolution of hydrogen bonds between proteins and membranes. CCD4a (red), CCD4b (green) and CCD4c (blue). Averaged H bond amounts are nine for CCD4a, seven for CCD4b and ten for CCD4c.

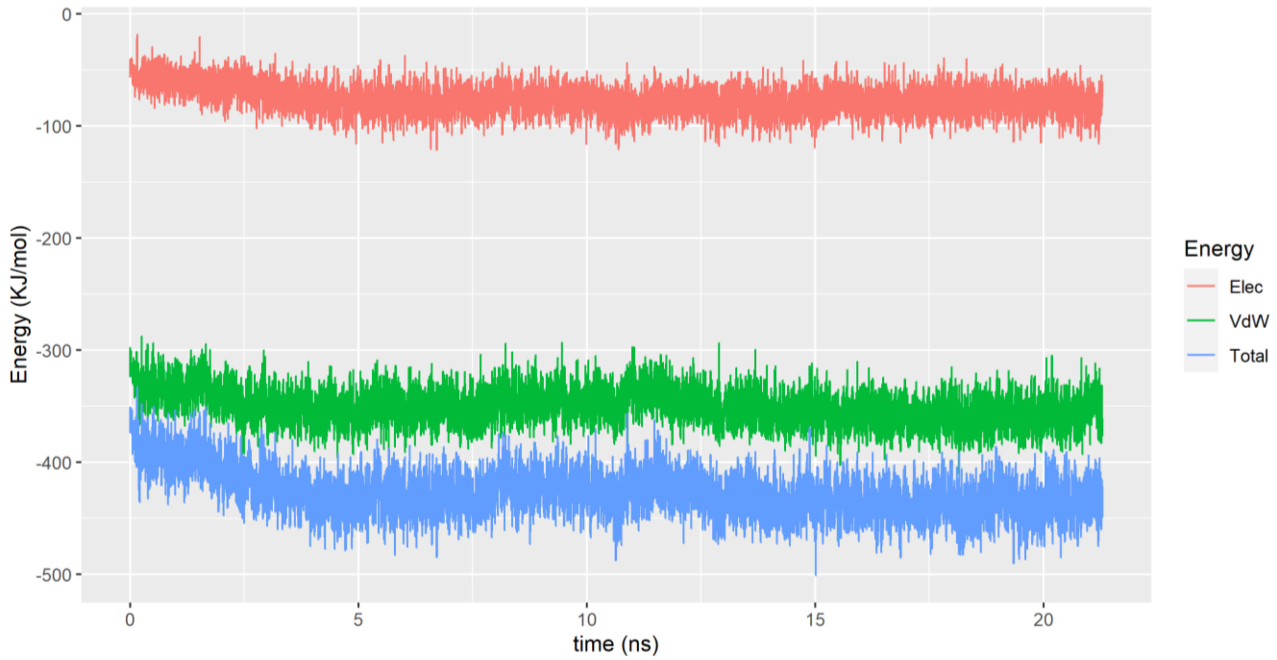

Figure S3. Evolution of the interaction energy of CCD4b in membrane with cryptoxanthin along the 20 ns molecular dynamics simulation trajectory. Electrostatic (red), van der Waals (green) and total (blue) potential energies are plotted.

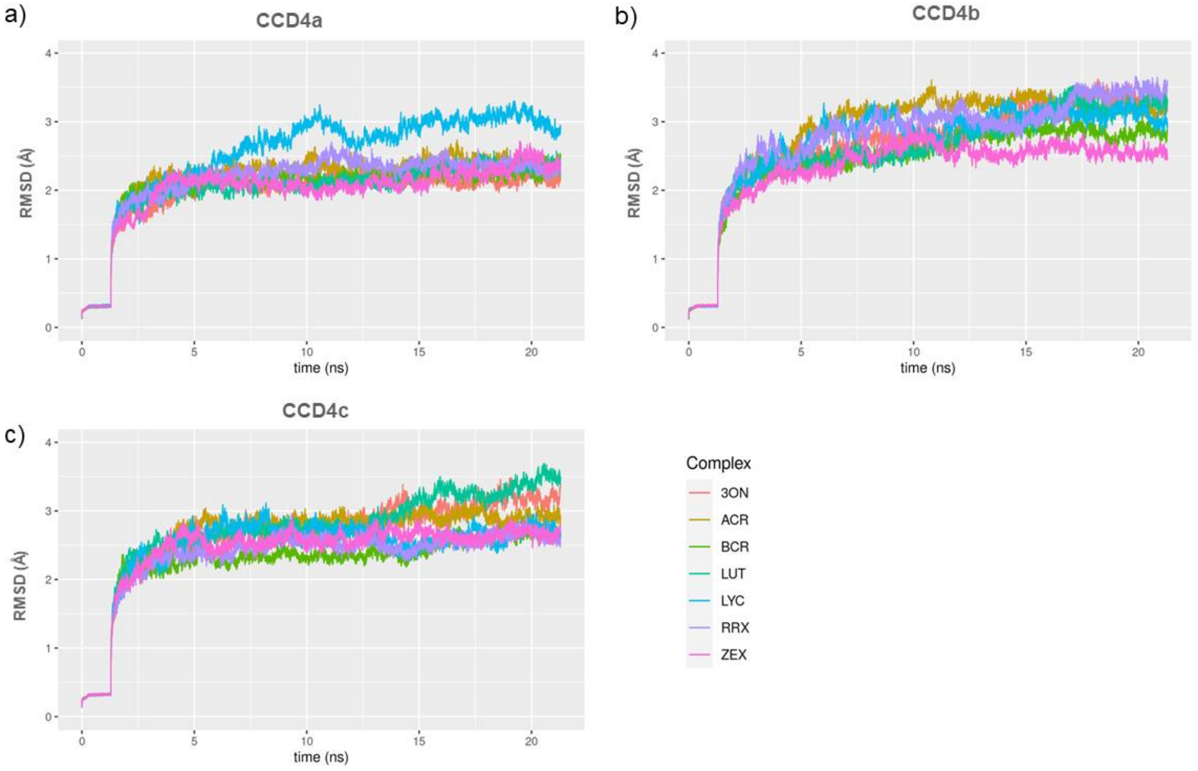

Figure S4. Values of RMSD as a function of the MD simulation time for the models of the complexes between CCD4a (a), CCD4b (b) and CCD4c (c) and seven carotenoid putative ligands. 3ON: 3-hydroxy-8'-apocarptenol, ACR: α-carotene; BCR: β-carotene; LUT: lutein; LYC: lycopene; RRX: cryptoxanthin: ZEX: zeaxanthin.

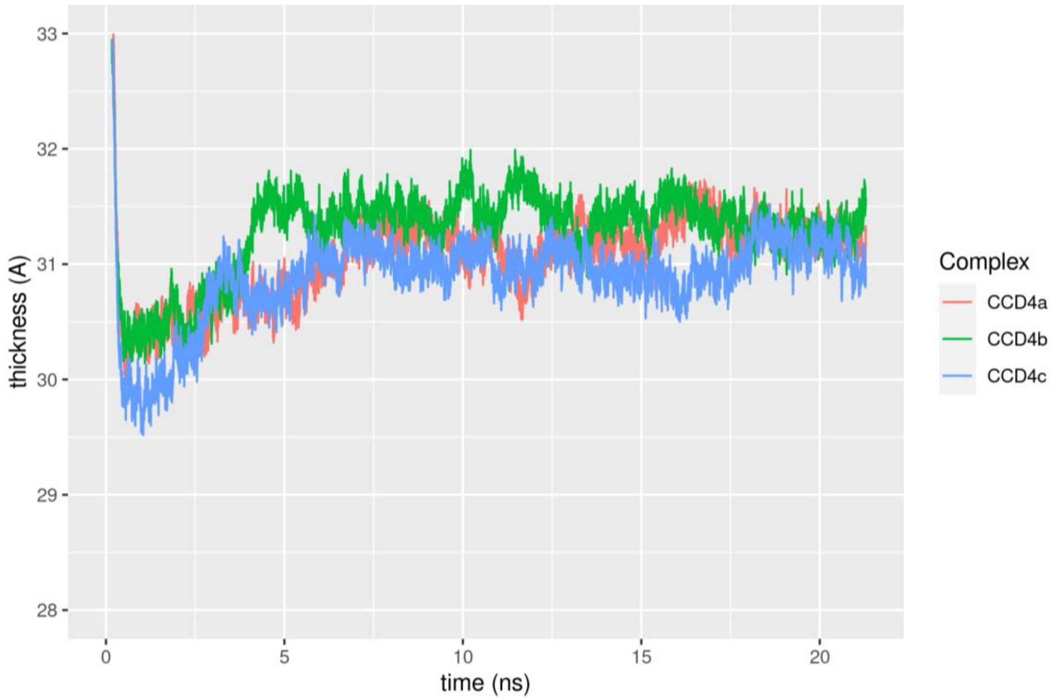

Figure S5. Membrane thickness for the three complexes throughout the simulations

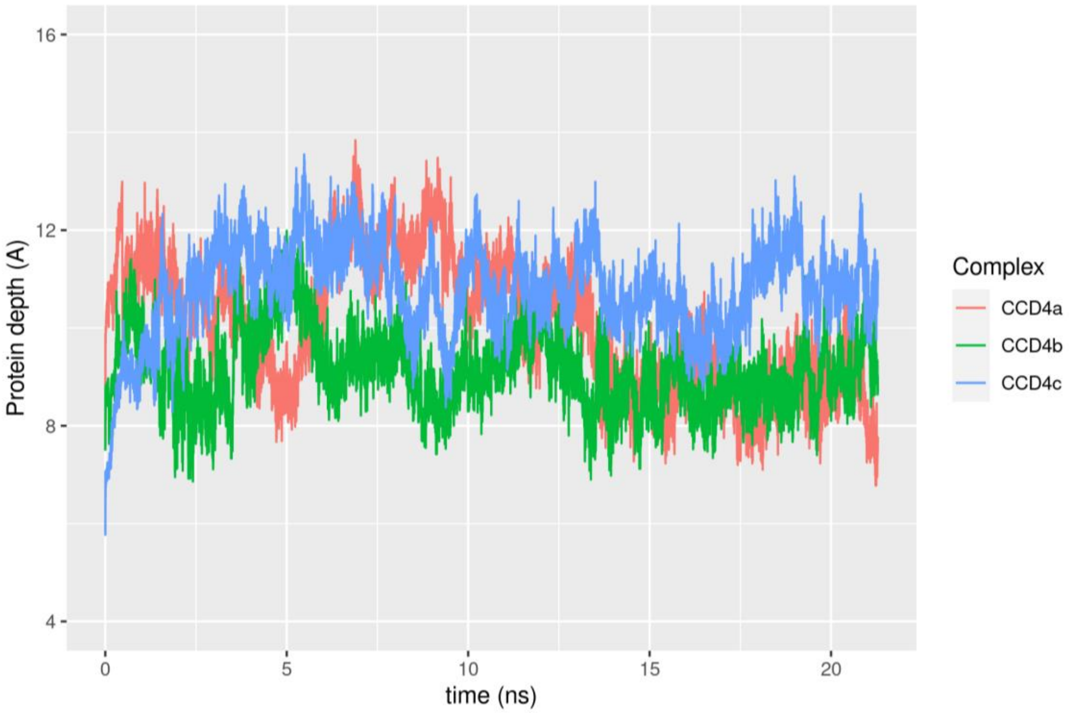

Figure S6. Depth of penetration of the protein into the membrane measured for the three studied complexes.

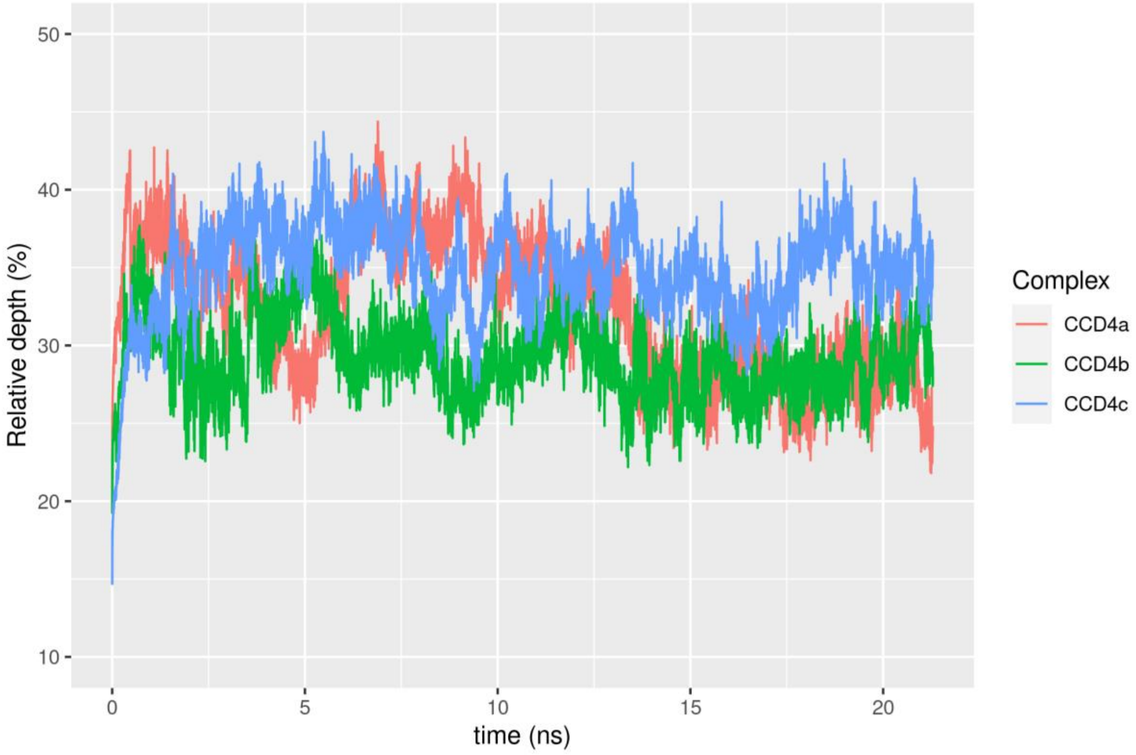

Figure S7. Relative membrane penetration of protein (RPD) evaluated through all simulations.

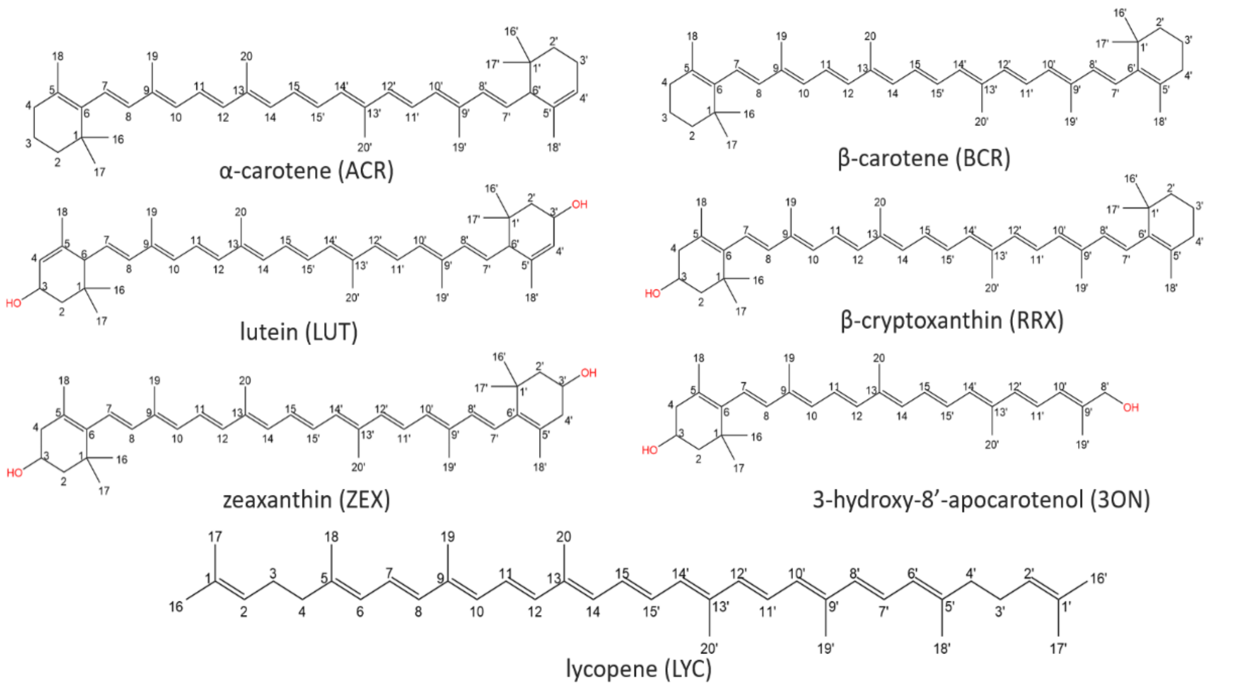

Figure S8: Structural formulas and numbering of apocarotenoid 3-hydroxy-8'-apocarptenol (3ON) and α-carotene, β-carotene, lutein, lycopene, β-cryptoxanthin and zeaxanthin carotenoids.
